# Multiple mechanisms shape FM sweep rate selectivity: complementary or redundant?

**DOI:** 10.3389/fncir.2012.00054

**Published:** 2012-08-17

**Authors:** Anthony J. Williams, Zoltan M. Fuzessery

**Affiliations:** Department of Zoology and Physiology, University of WyomingLaramie, WY, USA

**Keywords:** duration tuning, sideband inhibition, inferior colliculus

## Abstract

Auditory neurons in the inferior colliculus (IC) of the pallid bat have highly rate selective responses to downward frequency modulated (FM) sweeps attributable to the spectrotemporal pattern of their echolocation call (a brief FM pulse). Several mechanisms are known to shape FM rate selectivity within the pallid bat IC. Here we explore how two mechanisms, stimulus duration and high-frequency inhibition (HFI), can interact to shape FM rate selectivity within the same neuron. Results from extracellular recordings indicated that a derived duration-rate function (based on tonal response) was highly predictive of the shape of the FM rate response. Longpass duration selectivity for tones was predictive of slowpass rate selectivity for FM sweeps, both of which required long stimulus durations and remained intact following iontophoretic blockade of inhibitory input. Bandpass duration selectivity for tones, sensitive to only a narrow range of tone durations, was predictive of bandpass rate selectivity for FM sweeps. Conversion of the tone duration response from bandpass to longpass after blocking inhibition was coincident with a change in FM rate selectivity from bandpass to slowpass indicating an active inhibitory component to the formation of bandpass selectivity. Independent of the effect of duration tuning on FM rate selectivity, the presence of HFI acted as a fastpass FM rate filter by suppressing slow FM sweep rates. In cases where both mechanisms were present, both had to be eliminated, by removing inhibition, before bandpass FM rate selectivity was affected. It is unknown why the auditory system utilizes multiple mechanisms capable of shaping identical forms of FM rate selectivity though it may represent distinct but convergent modes of neural signaling directed at shaping response selectivity for important biologically relevant sounds.

## Introduction

The breakdown of complex sensory input into simpler components for extraction of specific cues that guide behavior is a common strategy utilized across sensory modalities. For the auditory system, this involves a reduction of complex sounds into their individual spectrotemporal components (Brugge, 1992). Many species exhibit a strong selectivity for the rate and direction of frequency modulations (FMs), which are common components of complex sounds (Pollak and Park, 1995; Andoni and Pollak, 2011). Several underlying mechanisms have been identified that shape FM selectivity involving the spectrotemporal integration of excitatory and inhibitory inputs across the receptive field of an auditory neuron (Suga, 1968; Britt and Starr, 1976; Heil et al., 1992; Shannon-Hartman et al., 1992; Gordon and O'Neill, 1998; Gittelman et al., 2009; Fuzessery et al., 2010). In the current study, we focus on how more than one mechanism can interact to shape the selectivity for FM sweep rate. The high-frequency region of the pallid bat inferior colliculus (IC) is ideal for this type of analysis due to a high degree of rate selectivity for the downward FM sweeps of their echolocation call (Brown, 1976; Bell, 1982; Fuzessery et al., 1993) and because the mechanisms driving FM rate selectivity have been identified (Fuzessery et al., 2010). Specifically, we focus on how duration tuning and sidebands of high-frequency inhibition (HFI) interact to shape rate selectivity for downward FM sweeps, and the neural circuitry that might shape these underlying mechanisms.

The IC is a highly integrative midbrain region of the auditory system that combines excitatory and inhibitory input from at least a dozen lower level nuclei (Pollak and Park, 1995; Oliver, 2005) and is also the first level of the auditory system where duration tuned neurons appear (Sayegh et al., 2011). The mechanisms driving duration tuning have typically been studied using simple stimuli such as pure tones (Ehrlich et al., 1997; Galazyuk and Feng, 1997; Chen, 1998; Fuzessery and Hall, 1999; Brand et al., 2000; Mora and Kossl, 2004; Jen and Wu, 2006; Perez-Gonzalez et al., 2006; Luo et al., 2008; Macias et al., 2011). Tone duration selectivity can be used to predict a neuron's best FM sweep rate, and it is likely that both forms of selectivity share the same underlying mechanisms (Fuzessery et al., 2006). Duration tuning has been described as the temporal integration of excitatory and inhibitory input using a variety of models, based on the results of both extra- and intracellular recording (Casseday et al., 1994; Fuzessery and Hall, 1999; Leary et al., 2008; Aubie et al., 2009; Sayegh et al., 2011).

Auditory neurons within the IC typically have a narrow excitatory receptive field bounded by sideband inhibition (Covey and Casseday, 1995; Ramachandran et al., 1999) that is responsible for much of the observed FM selectivity (Britt and Starr, 1976; Shannon-Hartman et al., 1992; Gordon and O'Neill, 1998; Koch and Grothe, 1998; Brimijoin and O'Neill, 2005; Williams and Fuzessery, 2011). In the pallid bat, many neurons exhibit an early-arriving (relative to excitation) band of low-frequency inhibition (LFI) that can shut down the response to an upward FM sweep and create selectivity for downward FM sweeps (Fuzessery et al., 2006; Razak and Fuzessery, 2006). The presence of a late-arriving band of HFI acts to suppress responses to slower sweep rates (Fuzessery et al., 2006; Razak and Fuzessery, 2006). It has also been shown that elimination of HFI, either through removing the high-frequency region from the FM sweep or by pharmacological blockade of inhibitory input, is associated with a subsequent loss of FM rate selectivity (Razak and Fuzessery, 2009; Williams and Fuzessery, 2011). However, given the complex integrative nature of the IC, many neurons exhibit more than one mechanism that can shape FM rate selectivity (Gordon and O'Neill, 1998; Fuzessery et al., 2010; Gittelman and Li, 2011). The focus of the current study was to evaluate the predictive value of duration tuning and HFI on FM rate selectivity, and how the two mechanisms interact to shape a neuron's selectivity for FM sweep rate.

## Materials and methods

Extracellular single-unit recordings were obtained from the IC of 44 adult pallid bats. Bats were captured in New Mexico and housed in a free-flight environmental chamber (85–90°F) maintained on a reverse 12:12 h light:dark cycle at the University of Wyoming Biological Sciences animal facility. The bats were fed mealworms raised on ground Purina rat chow. All surgical procedures, animal welfare assurances, and experimental manipulations were approved by an Institutional Animal Care and Use Committee based on guidelines required by the National Institutes of Health for animal research.

### Surgical procedures

Each bat was isolated from the main colony room and allowed 2–3 days to acclimate to their home cage prior to surgery. All surgical procedures were performed as previously described (Fuzessery et al., 2006) in a designated surgical suite. In brief, bats were initially sedated with an inhalation anesthetic (Isoflurane, USP) followed by an intraperitoneal injection of pentobarbital sodium (30 mg/kg of body weight) and acepromazine (2 mg/kg of body weight). Upon loss of reflexive responses to a toe pinch, animals were placed in a bite bar and a midline incision made in the scalp. The superficial muscles over the dorsal surface of the skull were carefully separated and reflected by blunt dissection. The anterior region of the skull was gently scraped clean and a thin layer of glass microbeads was applied and secured with cyanoacrylate for placement of a head pin. A 1 mm^2^ exposure was made over the left or right IC by carefully excising the skull with a microscalpel. Exposed muscle was covered with petroleum jelly (Vaseline®) and the skull was kept moist with periodic applications of physiological saline throughout the course of the recording session. Following surgery, the animals were taken to the recording chamber (see below) and secured in a Plexiglas restraining device. A cylindrical aluminum head pin was mounted to a cross bar and secured to the anterior skull using dental cement to prevent movement of the head.

### Recording and data acquisition procedures

Bats were isolated in a heated (85–90°F), sound-proofed chamber lined with anechoic foam during the 6–8 h recording session. Auditory stimuli were generated by digital hardware (Modular Instruments and Tucker Davis Technologies) controlled by a custom-written software program (Fuzessery et al., 1991). Modulated waveforms were amplified with a stereo amplifier and presented as monaural closed-field stimuli through Infinity emit-K ribbon tweeters fitted with funnels attached for insertion into the pinnae. Speaker output was calibrated with a Bruel and Kjaer 1/8 inch microphone placed at the tip of funnel (±15 dB response from 20 to 70 kHz).

*In vivo* single-unit recordings of extracellular neuronal activity were obtained with glass microelectrodes (1 M NaCl, 2–5 MΩ resistance) mounted diagonally in a “piggy-back” configuration (Havey and Caspary, 1980) to a five-barrel glass pipette (WPI) used for iontophoresis of inhibitory receptor antagonists (see below). All data were recorded from the high-frequency region of the pallid bat IC (best frequency = 30–60 kHz) at penetration depths of 1000–2000 μm from the surface of the brain using a similar recording protocol as described previously (Razak and Fuzessery, 2009; Williams and Fuzessery, 2011).

The best frequency and excitatory tuning curve were determined with single tones over a range of intensity levels. Rise/fall times were 1 ms unless signals were shorter than 1 ms, in which case they were each one half of the signal. All subsequent recordings were performed at a single intensity level 5–10 dB above the intensity threshold for the best frequency. Pairs of tones, offset in time, from within and outside the excitatory bandwidth, were used to determine the extent of sideband inhibition, using the two-tone inhibition protocol described below. FM sweeps (30 kHz bandwidth) and best frequency tones were then presented over a range of durations (0.1–100 ms) to establish, respectively, selectivity for FM rate and tone duration. Response magnitudes for stimuli are defined as the total number of spikes in response to 30 stimulus presentations presented at an interpulse interval of 400 ms. Single-unit output was identified audiovisually, and based on the consistency of the action potential waveform, and on high signal-to-noise ratio.

### Predicted rate functions

The predicted rate function of a neuron was derived from its response to a single tone presented over a range of tone durations (Figure 2). We have previously (Fuzessery et al., 2006) predicted the best sweep rate of neurons with bandpass duration selectivity by dividing the bandwidth (kHz) of the excitatory tuning curve by the best duration (ms). This is the sweep rate at which an FM sweep will traverse the excitatory tuning curve in a time equal to the neuron's best duration. In the present study, we predict the entire rate function of a neuron using the responses to a single excitatory tone over a range of durations to predict the response at a given sweep rate. Sweep rate selectivity was predicted from the tone by dividing the bandwidth of the tuning curve by the duration of the tone. The response to that tone duration was then used to construct the sweep rate function. For example, the neuron in Figure 2 had an excitatory bandwidth of 13 kHz. Therefore, a 15 ms tone duration (Figure 2A, arrow) would correspond to a 0.87 kHz/ms sweep rate. The response at this duration was 35 spikes, and was used to predicted the response at 0.87 kHz/ms (Figure 2B, arrow). This process was repeated for each data point in the duration function (Figure 2A) to construct the entire predicted sweep rate function, which was then compared with the actual rate function (Figure 2B).

### Two-tone inhibition

The spectral width and relative arrival time of inhibitory sidebands were determined using a two-tone inhibition over time protocol as previously described (Fuzessery et al., 2006). The focus of the present study is HFI, which if present in this population of neurons, always arrives later than excitation, and serves to determine the slowest sweep rate to which a neuron will respond.

In brief, inhibitory tones from the high-frequency region (if present) were paired with an excitatory tone of the same intensity level to audiovisually map out the frequency range of sideband inhibition. Excitatory tones were always shorter than inhibitory tones, with excitatory tones of 1–2 ms duration, and inhibitory tones of 5–10 ms duration. The delay between an excitatory and inhibitory tone was then varied to determine the relative arrival time of inhibition for each sideband region. Specifically, the delay between the onsets of the two tones was varied to determine the delay-frequency combination resulting in at least a 90% reduction in the response. If the inhibitory tone had to be presented before the excitatory tone for suppression to occur (i.e., positive delay) then excitatory input was assumed to arrive before inhibition. If the inhibitory tone could suppress the excitatory tone even when presented after the excitatory tone (i.e., negative delay) then inhibitory input was assumed to arrive before excitation. A predicted FM cutoff rate (i.e., the slowest rate to which the neuron would respond) was calculated from the bandwidth and arrival time of HFI as previously described (Fuzessery et al., 2006) using the following formula: predicted FM cutoff rate (kHz/ms) = HFI bandwidth (kHz)/HFI arrival time (ms). FM sweeps presented at a rate slower than the predicted FM cutoff rate would cause HFI to arrive before excitation, and the response would be suppressed.

### Microiontophoresis

Microiontophoretic applications of inhibitory receptor blockers were delivered using a previously described protocol (Razak and Fuzessery, 2009; Williams and Fuzessery, 2011). Immediately before a recording session, individual iontophoresis barrels were loaded with gabazine (GBZ, 10 mM, pH 4.0, Sigma) or strychnine (STRYCH, 3 mM, pH 4.0, Sigma) dissolved in physiological saline. The center barrel was used as a balance electrode (1 M sodium chloride). A retaining current (−15 nA) was used to retain the drugs during the search phase and pre-drug (control) recording phase. Escalating iontophoretic ejection currents of +10 to +60 nA were used to apply the drug. Three types of tests were performed following drug application to confirm the effectiveness of inhibitory receptor blockade, as previously described (Razak and Fuzessery, 2009; Williams and Fuzessery, 2011) (1) Recovery data, quantified as number of spikes, were obtained in 25 neurons at 5 min intervals after the ejection current was turned off to monitor the return to pre-drug response levels, which took 10–30 min. (2) Current was passed through the balance barrel in 18 neurons to verify that the ejection current did not affect the response. (3) In all neurons, ejection currents were gradually increased from +10 to +60 nA, with responses monitored at each interval, to avoid possible response saturation. When both drugs were tested on a neuron, the response of the neuron returned to pre-drug levels before the second drug was tested.

### Data analysis

A Pearson correlation test was used to compare predicted versus actual FM rate responses. A Fisher Exact Test was used to evaluate the proportion of neurons affected by the application of GBZ or STRYCH on duration tuning or FM rate selectivity. All data are presented as the mean ± S.D. *P*-values <0.05 were considered significant.

## Results

**Figure 1:**
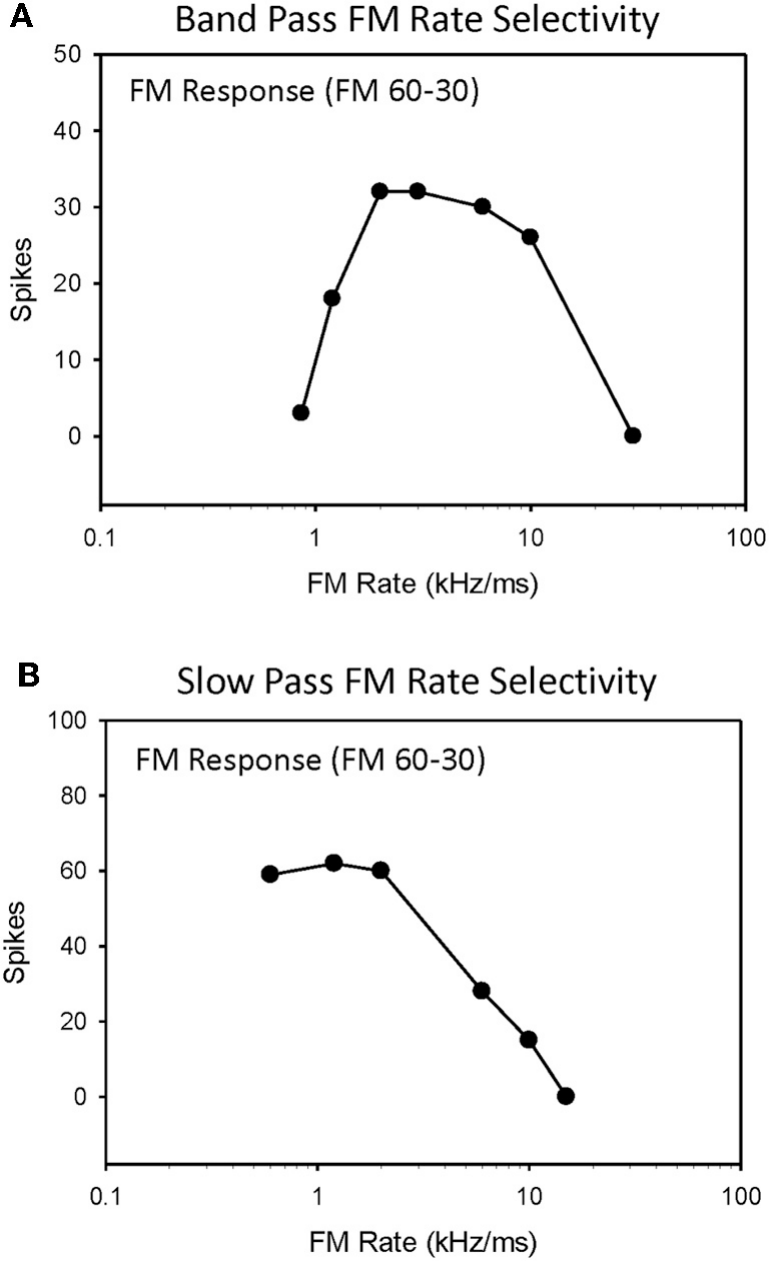
**Single-unit responses from the high-frequency region of the pallid bat IC were recorded for downward FM sweep rates ranging from 0.3 to 300 kHz/ms and classified as either bandpass (A) or slowpass (B)**.

Selectivity for downward FM sweep rates between 0.3–300 kHz/ms (30 kHz bandwidth, 0.1–100 ms duration) were measured in 79 IC neurons, and the roles of duration tuning and HFI in shaping their selectivity were evaluated (Table 1). The majority of neurons evaluated (72%, 57/79) were tuned to a limited range of FM sweep rates, and exhibited bandpass rate selectivity (Figure 1A). The remaining neurons (28%, 22/79) were not tuned to sweep rates, and instead exhibited slowpass rate selectivity (Figure 1B). This percentage of rate-tuned neurons is similar to that reported in previous studies of the pallid bat IC (Fuzessery et al., 2010). We have previously reported the presence of fastpass rate selectivity (Fuzessery et al., 2006), but because, in the present study, we extended the range of sweep rates to include faster rates (300 kHz/ms), all neurons eventually ceased to respond as sweep rates were increased.

**Table 1:** **Comparison of FM and tonal stimulus selectivity to presence or absence of HFI**.

**FM rate**	**Bandpass duration**	**HFI**	**Percent**
Slowpass (*n* = 22)	No	No	100% (22/22)
Bandpass (*n* = 57)	No	Yes	62% (35/57)
	Yes	No	14% (8/57)
	Yes	Yes	24% (14/57)

Numbers in parentheses indicate the number of neurons in each group/total number of either slowpass or bandpass FM selective neurons.

Table 1 summarizes the four possible conditions in terms of the mechanisms shaping sweep rate selectivity. If neither duration tuning or HFI was present, the neurons all had slowpass rate functions (Figure 1B). If either or both duration tuning were present, the neurons had bandpass rate functions (Figure 1A). When both mechanisms were present, their relative contributions to shaping rate selectivity were determined by either eliminating HFI by starting the downward FM sweep at a frequency just lower than the high-frequency inhibitory sideband, or by eliminating duration selectivity or HFI through the iontophoresis of inhibitory receptor blockade (Table 2).

**Table 2:** **Effect of eliminating the underlying mechanisms (duration tuning/HFI) on bandpass FM rate selectivity**.

**Bandpass duration tuning**	**HFI**	**Bandpass FM rate selectivity**	***n***	**Test**
Eliminated	Absent	Eliminated	2	Iontophoresis (2 STRYCH)
Intact	Absent	Intact	3	Iontophoresis (2 STRYCH, 1 GBZ)
Eliminated	Intact	Intact	4	Iontophoresis (2 STRYCH, 2 GBZ)
Eliminated	Eliminated	Eliminated	1	Iontophoresis (1 STRYCH)
Intact	Intact	Intact	5	Iontophoresis (1 STRYCH, 4 GBZ)
Intact	Eliminated	Intact	5	Removal of HFI from FM Sweep

Two types of tests were used (right column) to evaluate the removal of duration tuning and HFI: (1) iontophoresis of GBZ or STRYCH to block inhibitory input or (2) directly removing HFI by excluding the high-frequency region from the FM sweep. “Absent” indicates that HFI was not present. “Eliminated” responses indicate a conversion of duration tuning from bandpass to longpass or conversion of FM rate tuning from bandpass to slowpass. “Intact” responses indicate that bandpass selectivity did not change.

### Predicting sweep rate selectivity from duration tuning

**Figure 2:**
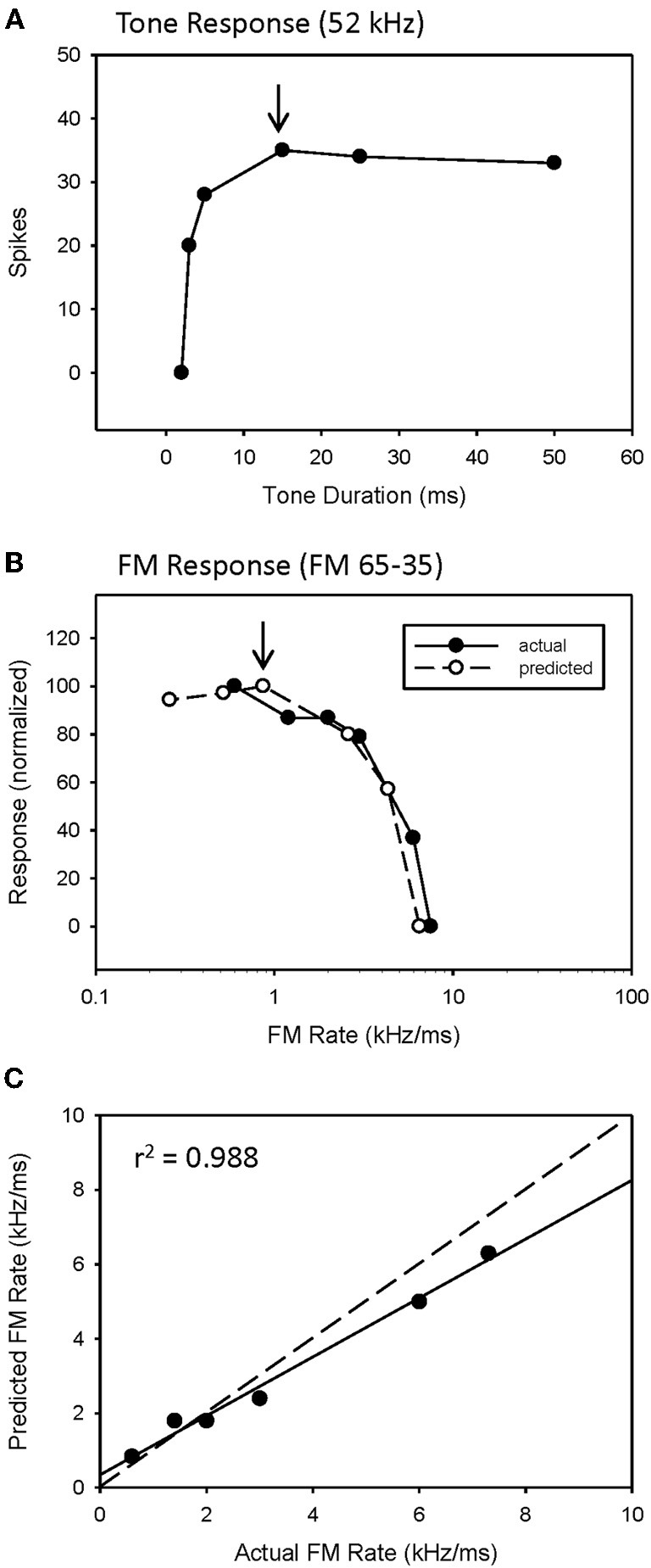
**A slowpass FM rate selective neuron (B) exhibiting longpass duration tuning for tones (A).** The duration function, based on longpass duration tuning (see Methods), predicted the slowpass FM rate response **(B)** with a significant correlation between predicted and actual FM rates **(C)**. The dashed line indicates perfect predictions, and the solid line indicates the actual correlation.

We have previously (Fuzessery et al., 2006) predicted only the best sweep rates of neurons with bandpass rate selectivity from their duration functions, by dividing the bandwidth of their excitatory tuning curves (kHz) by their best durations (ms). This gives the sweep rate at which an FM sweep will pass through the excitatory tuning curve in a time equal to the best duration. Here we construct the entire predicted rate function for each neuron from its duration function. Figure 2 demonstrates how the duration function was used to predict the FM rate response. This neuron exhibited a longpass duration selectivity, responding to tones longer than 3 ms (Figure 2A). It lacked HFI, so, as expected, it exhibited a slowpass rate selectivity, responding to sweep rates slower than 6 kHz/ms (Figure 2B). Its predicted rate function was constructed from the duration function by dividing the width of the excitatory tuning curve by the tone duration, and using the response magnitude at that calculated rate to create a predicted sweep rate function. This neuron had a tuning curve width of 13 kHz. To illustrate the construction of one point in the predicted function (arrows, Figures 2A,B), a 15 ms tone duration is equivalent to the time taken for a sweep rate of 0.87 kHz/ms (13 kHz/15 ms) to traverse the tuning curve. The response at this tone duration was 36 spikes (Figure 2A), and when normalized in the predicted rate function, was 100% maximum response (Figure 2B). The predicted rate function (Figure 2B) of this neuron was strongly predictive of the actual FM rate function (Figure 2C). Overall, there was a positive correlation observed between predicted and actual FM rates of all slowpass neurons evaluated (mean *r*^2^ = 0.936 ± 0.065, *n* = 22).

**Figure 3:**
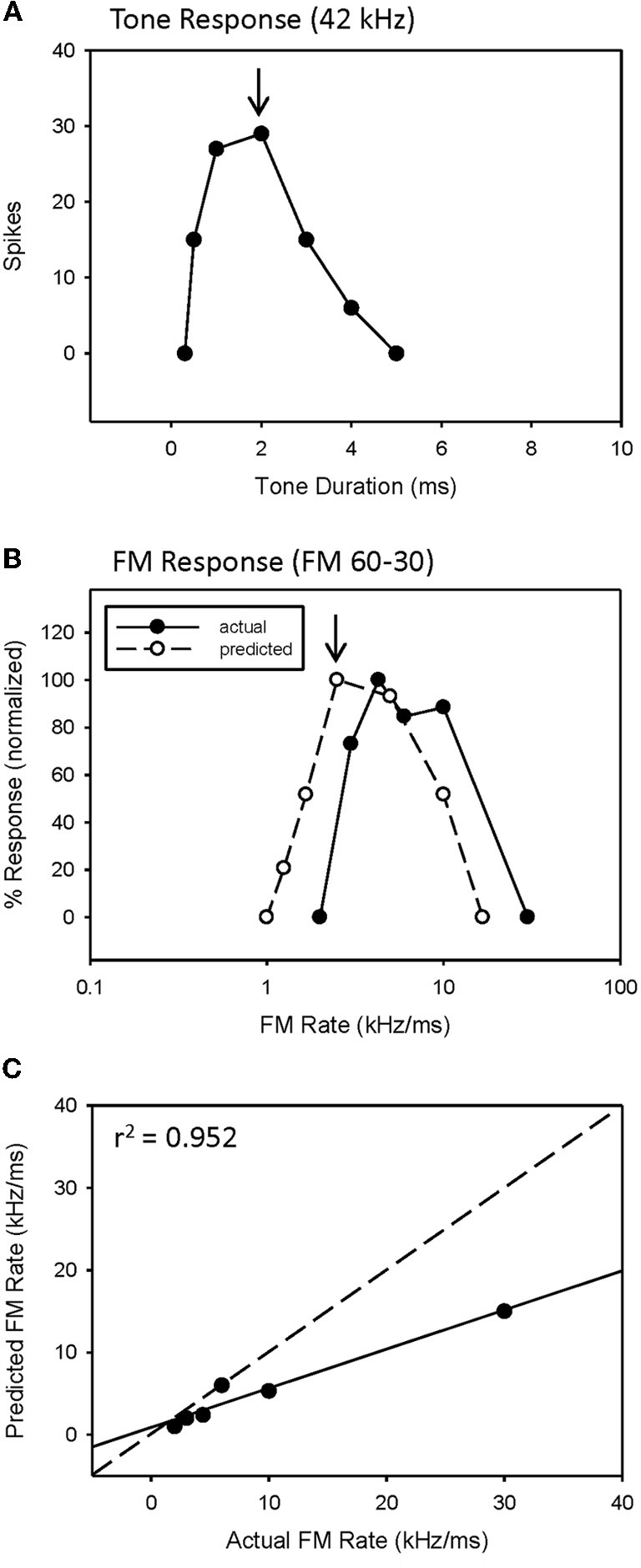
**The duration function of a bandpass duration-tuned neuron (A) that was predictive of a bandpass FM rate response (B) with a significant correlation (C) between predicted and actual FM rates (B)**.

An example of a predicted rate function for a neuron with a bandpass duration function, and lacking HFI, is shown in Figure 3. This neuron had an excitatory tuning curve bandwidth of 5 kHz, so the maximum response to a 2 ms tone (Figure 3A, arrow) predicts the response to a 2.5 kHz/ms sweep rate (Figure 3B, arrow), with a high correlation between predicted and actual FM rates (Figure 3C, *r*^2^ = 0.952, *p* < 0.05). Overall, a positive correlation between predicted and actual rate functions was observed for all 8 bandpass FM rate selective neurons tested that did not exhibit HFI (Table 1, mean *r*^2^ = 0.878 ± 0.178, *n* = 8).

However, while the correlations were high, the absolute values for actual and predicted functions were off in a consistent manner. Note the shift in the predicted function toward slower sweep rates, relative to the actual FM rate response (Figure 3B). This is reflected in the shallower slope of the prediction line (Figure 3C, slope = 0.476). This was a typical pattern observed between the predicted and actual rate functions across the entire population of neurons tested, both slowpass and bandpass. Although some neurons did exhibit both high correlation and slope values (e.g., Figure 2C), the average difference in slopes between predicted and actual FM rates = 0.617 ± 0.373 (*n* = 48), indicating a 1.6 fold (1/slope) underestimation of actual FM responses on average. As will be discussed, this shift in the predicted rate function toward slower rates is likely due to an underestimation of the excitatory bandwidth, which is broader when measured with FM sweeps as opposed to tones.

### Interaction of duration tuning and HFI on FM rate selectivity

While the entire sweep rate function of a neuron can be predicted from its tone duration function, HFI can be used to predict only the slowest sweep to which a neuron will respond, which is the bandwidth of HFI (kHz) divided by the arrival time (ms) of HFI relative to the arrival of excitation (Fuzessery et al., 2006). This is the cutoff rate, i.e., the sweep rate at which the delayed HFI will arrive at the same time as the excitatory input, and suppress the response.

The majority of bandpass FM rate-selective neurons exhibited HFI (86%) and either longpass (62%) or bandpass (38%) duration tuning for tones (Table 1). It is predicted that those with longpass duration functions will have bandpass rate selectivity shaped entirely by HFI, while those with bandpass duration functions will have bandpass rate selectivity shaped by either mechanism, or a combination of the two.

**Figure 4:**
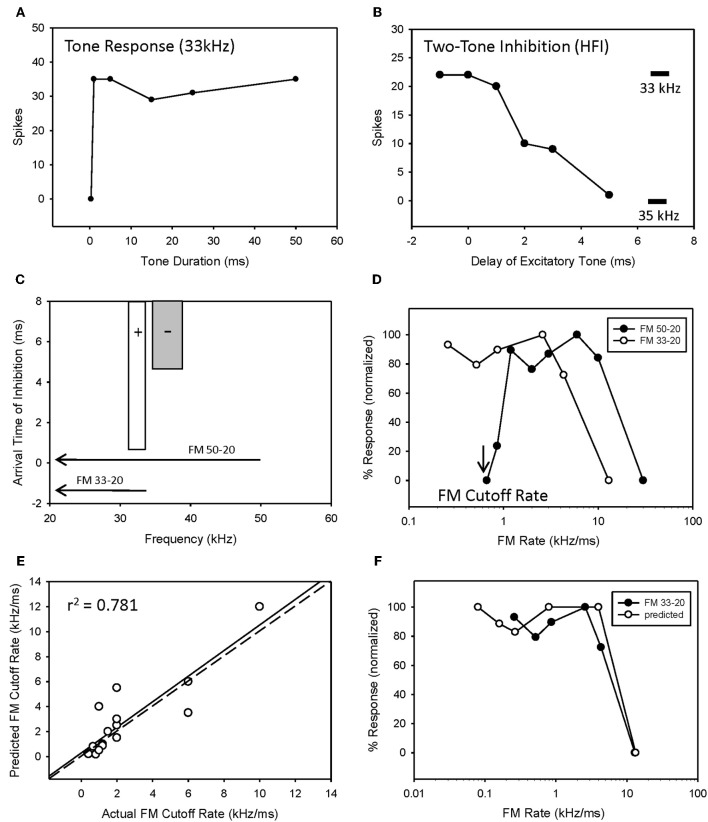
**HFI acts as a fastpass FM rate filter.** Neuron exhibiting longpass duration tuning for tones **(A)** and a delayed HFI **(B)**. FM sweep responses were compared both with and without the contribution of HFI **(C)**. Removal of HFI converted the FM rate response from bandpass to slowpass **(D)**. A positive correlation was observed between predicted and actual FM cutoff rates **(E)** as derived from the bandwidth and arrival time of HFI (see Methods). The duration function was only predictive of the FM rate response when HFI was removed from the sweep **(F)**.

The majority of bandpass FM rate selective neurons exhibited longpass duration tuning and HFI (Table 1). It would be expected that the removal of HFI, by starting a downward sweep at a frequency just below this inhibitory domain, would eliminate their bandpass selectivity. This was tested in 13 of these neurons. An example is shown in Figure 4. This neuron exhibited longpass duration tuning for tones with a strong response to tone durations >1 ms (Figure 4A). A tone (35 kHz) from within high-frequency inhibitory sideband (Figure 4C) completely suppressed the response to a best-frequency tone (33 kHz) when the higher frequency tone was presented 5 ms before the excitatory tone (Figure 4B). Elimination of HFI in the downward 33–20 kHz sweep (Figure 4C) sweep converted the FM rate response from bandpass to slowpass (Figure 4D) demonstrating that HFI acts as a fastpass filter for FM rate selectivity by suppressing slow FM sweep rates. In this neuron, the predicted FM cutoff rate was 0.8 kHz/ms (4 kHz/5 ms), which was close to the actual FM cutoff rate of 0.67 kHz/ms (Figure 4D).

Overall, there was a positive correlation between predicted and actual FM cutoff rates (Figure 4E, *r*^2^ = 0.781, *n* = 17, *P* < 0.05). Moreover, the predicted slowpass rate functions from duration tuning were accurate when the HFI was removed from the sweep, as demonstrated in Figure 4F (*r*^2^ = 0.975 between actual and predicted rates, *p* < 0.05). Thus, when bandpass rate selective neurons had longpass duration functions and HFI, their rate selectivity was shaped entirely by HFI.

**Figure 5:**
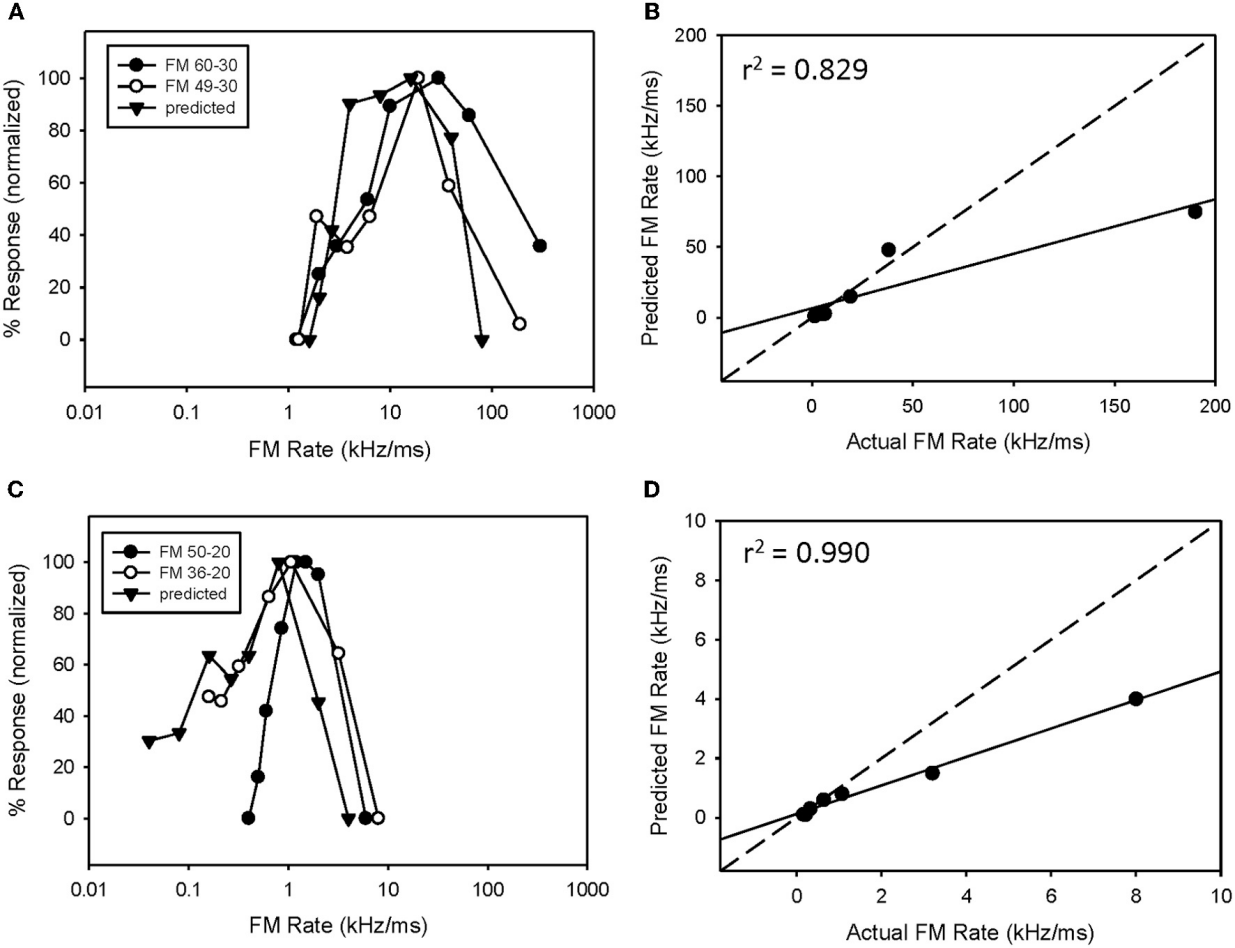
**In neurons expressing HFI, predicted responses were compared to FM sweeps both in the presence and absence of HFI by eliminating the high-frequency region from the sweep.** The effect of removing HFI ranged from a minimal change in the FM rate response **(A)**, a broadening of the FM rate response to include slower FM sweep rates **(C)**. In each case, predicted and actual FM rates were significantly correlated but only in the absence of HFI **(B,D)**.

Fourteen neurons (24%) expressed both bandpass duration tuning for tones and HFI. The effect of removing HFI from a downward FM sweep was tested in five of these neurons. Effects ranged from a minimal change in FM selectivity (Figure 5A) to a broadening of the bandpass rate selectivity to include slower FM sweep rates (Figure 5C). In the first case (Figure 5A), this can be interpreted as HFI setting a cutoff rate that was the same or lower than that shaped by the bandpass duration function; hence eliminating HFI had little effect on the rate function. Moreover, the predicted rate function accurately predicted the actual rate function, with or without HFI in the FM sweep. In contrast, in the second case (Figure 5C), the cutoff rate shaped by HFI occurred at a faster rate than that shaped by bandpass duration tuning. Therefore, the bandpass rate function broadened when HFI was removed, and was then accurately predicted by the duration function. In all cases, bandpass FM rate selectivity remained intact and a high correlation was observed between predicted and actual FM rate responses, when HFI was excluded from the FM sweep (Figures 5B,D, mean *r*^2^ = 0.948 ± 0.068, *n* = 5). Thus, when both HFI and bandpass duration tuning were present, duration tuning could either shape the entire rate function, or HFI could determine the cutoff rate, and duration tuning determine the reminder of the rate function.

Despite the fact that FM sweep rates were tested over a broad range of 0.3–300 kHz/ms, three neurons were responsive to even slower rates, and their bandpass duration functions predicted that, if slower rates had been tested, these neurons would have exhibited bandpass rate selectivity. Because it was not clear whether their predicted and actual rate functions were similar, these neurons were excluded from the study.

### Inhibitory mechanisms shaping FM rate selectivity

**Figure 6:**
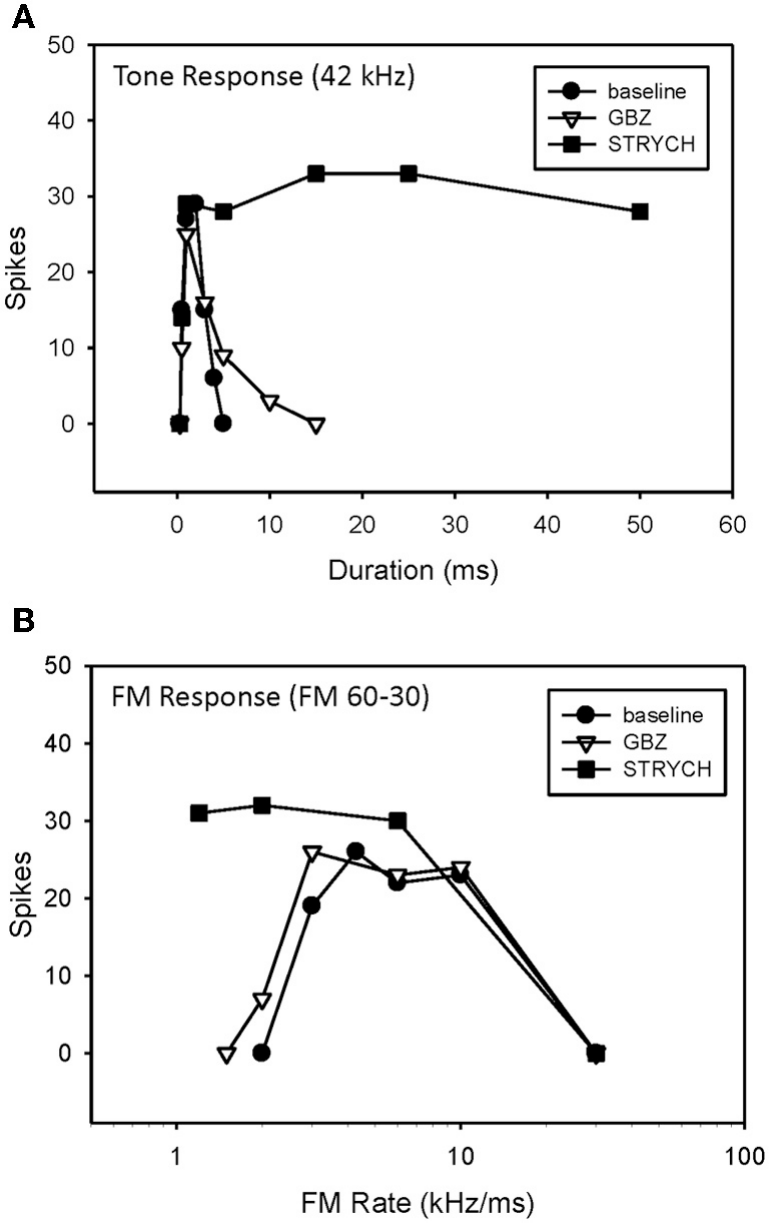
**Conversion of duration tuning (A) and FM rate selectivity (B) from bandpass to slowpass following application of STRYCH.** GBZ had no effect on either parameter.

**Figure 7:**
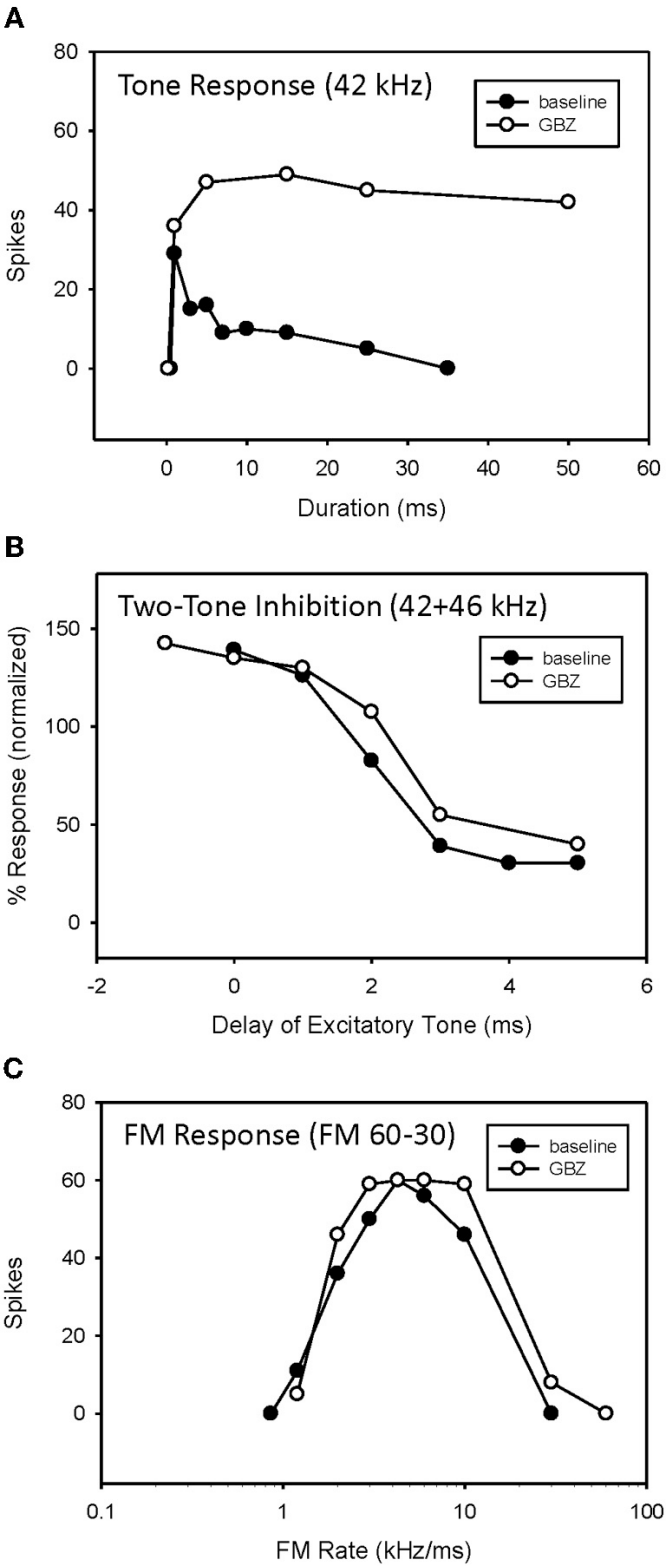
**Application of GBZ converted duration tuning from bandpass to longpass (A) but did not eliminate HFI (B) or bandpass FM rate selectivity (C) in this neuron**.

A previous study demonstrated that, in the majority of neurons tested, bandpass duration tuning is created at the level of the IC in the pallid bat by an on-best frequency inhibition and can be eliminated by blocking GABAergic input with bicuculline (Fuzessery and Hall, 1999). In the present study, bandpass duration tuning was eliminated following application of either STRYCH (Figure 6A, 5/7 neurons) or GBZ (Figure 7A, 3/8 neurons), indicating that either inhibitory pathway (glycinergic or GABAergic) is involved in sculpting this form of selectivity.

The iontophoretic blockade of inhibitory inputs was used as a second test of the roles of duration tuning and HFI in shaping rate selectivity. Table 2 summarizes the effects of inhibitory receptor blockade on 15 bandpass duration tuned neurons, some of which also had HFI, and its subsequent effect on bandpass rate tuning. In the first two rows, the neurons lacked HFI. If receptor blockade eliminated duration tuning, rate tuning was also eliminated. In the next three rows, neurons possessed both HFI and duration tuning. Both mechanisms had to be eliminated by receptor blockade to eliminate rate tuning. If one mechanism was not eliminated, rate tuning remained intact. For neurons in the bottom row, HFI was eliminated not by iontophoresis, but rather by eliminating HFI by starting the downward sweep at a frequency lower than the inhibitory sideband (e.g., Figure 4C). Since duration tuning remained intact, so did the rate tuning.

Overall, when both underlying mechanisms were absent or eliminated, bandpass rate selectivity was lost (3/20 neurons), but if either of the mechanisms remained intact, bandpass FM rate selectivity also remained intact (17/20 neurons) (*P* < 0.05 between groups, Fisher's Exact Test).

An example of a bandpass rate-selective neuron expressing only bandpass duration tuning is shown in Figure 6. Blocking GABAergic input had only a minor effect on its bandpass duration tuning (Figure 6A) and consequently a minor effect on rate selectivity, which remained bandpass (Figure 6B). In contrast, blocking glycinergic input changed its duration function from bandpass to longpass (Figure 6A), and its rate function from bandpass to slowpass (Figure 6B).

An example of bandpass rate-selective neuron expressing both duration tuning and HFI is show in Figure 7. As in the previous neuron, blocking inhibition, in this case GABAergic inhibition, eliminated bandpass duration tuning (Figure 7A). This neuron's HFI inhibition was examined by delaying an excitatory tone (42 kHz) with respect to a high-frequency inhibitory tone (46 kHz), and showed that the arrival of inhibition was delayed by about 3 ms (Figure 7B). Blocking inhibition had little effect on the two-tone inhibition function. Since HFI remained intact after blocking GABAergic inhibition, the neuron's bandpass rate selectivity also showed little change before and during receptor blockade (Figure 7C).

**Figure 8:**
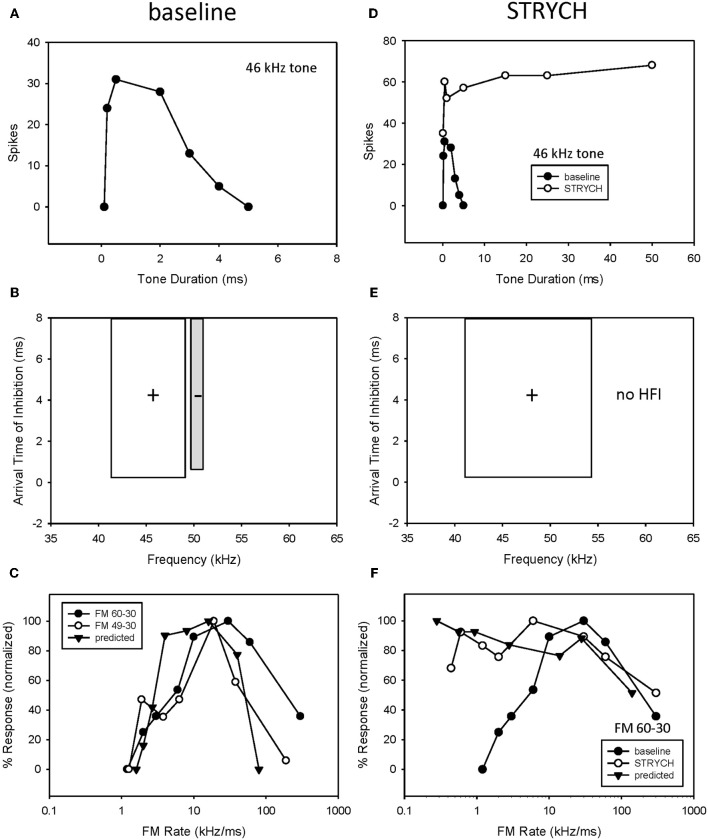
**If more than one mechanism was present both had to be eliminated before FM rate selectivity was affected.** In this neuron, exhibiting both bandpass duration tuning **(A)** and HFI **(B)**, the elimination of HFI from the FM sweep did not eliminate bandpass FM rate selectivity **(C)**. In comparison, application of STRYCH eliminated bandpass duration tuning **(D)**, HFI **(E)**, and bandpass FM rate selectivity **(F)**.

A third example shows the result of eliminating both underlying mechanisms (Figure 8). Blocking glycinergic input eliminated both bandpass duration tuning (Figures 8A,D) and HFI (Figures 8B,E). Following the elimination of HFI, the bandwidth of the excitatory tuning curve expands to well beyond the frequencies in the inhibitory sideband (Figure 8E). Prior to blocking inhibition (Figure 8C), the elimination of HFI with a downward sweep (49–30 kHz) had no effect on the rate function, indicating that the neuron's duration tuning played the dominant role in shaping rate tuning. When STRYCH was applied, this bandpass duration tuning was eliminated, and, as expected, so was the bandpass rate selectivity. Moreover, the duration function during disinhibition predicted the rate selectivity (Figure 8F).

**Figure 9:**
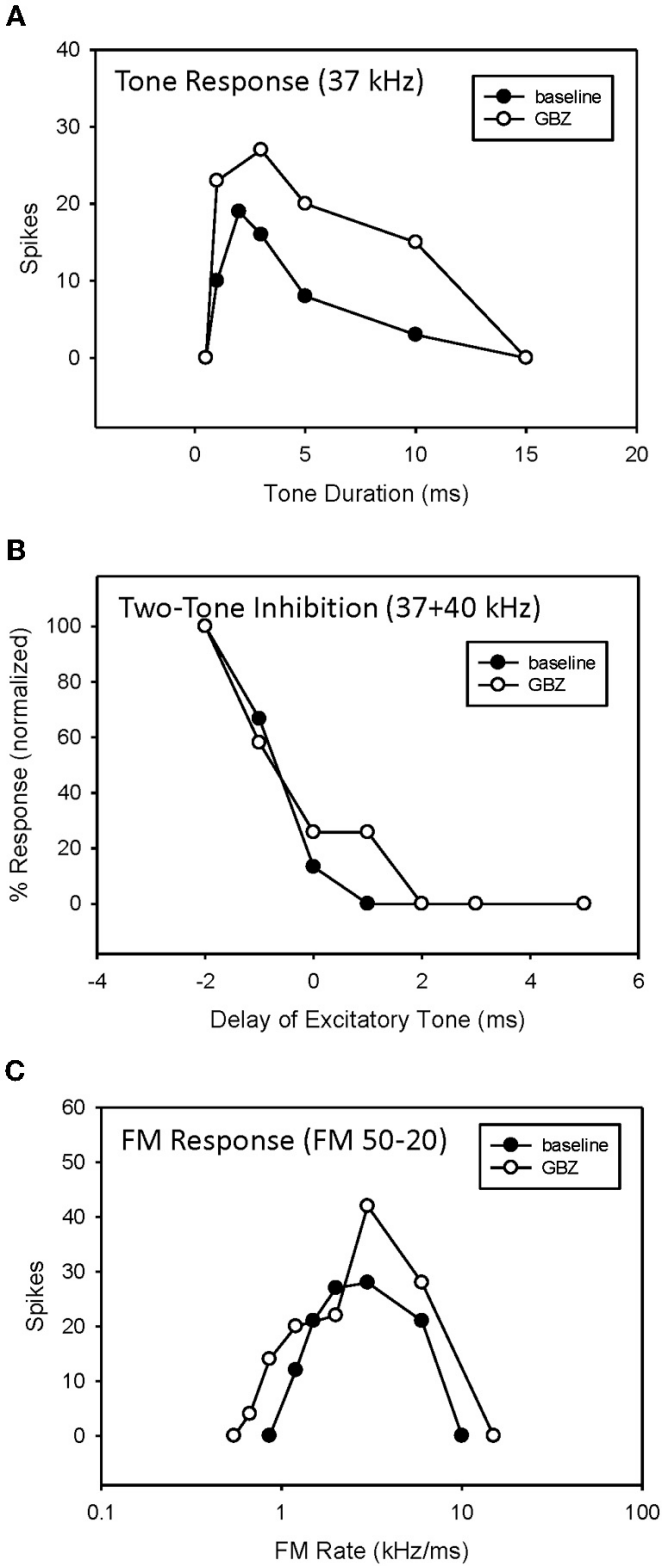
**Application of GBZ was ineffective at eliminating bandpass duration tuning (A), HFI (B), or band-pass FM rate selectivity (C) in this neuron**.

**Figure 10:**
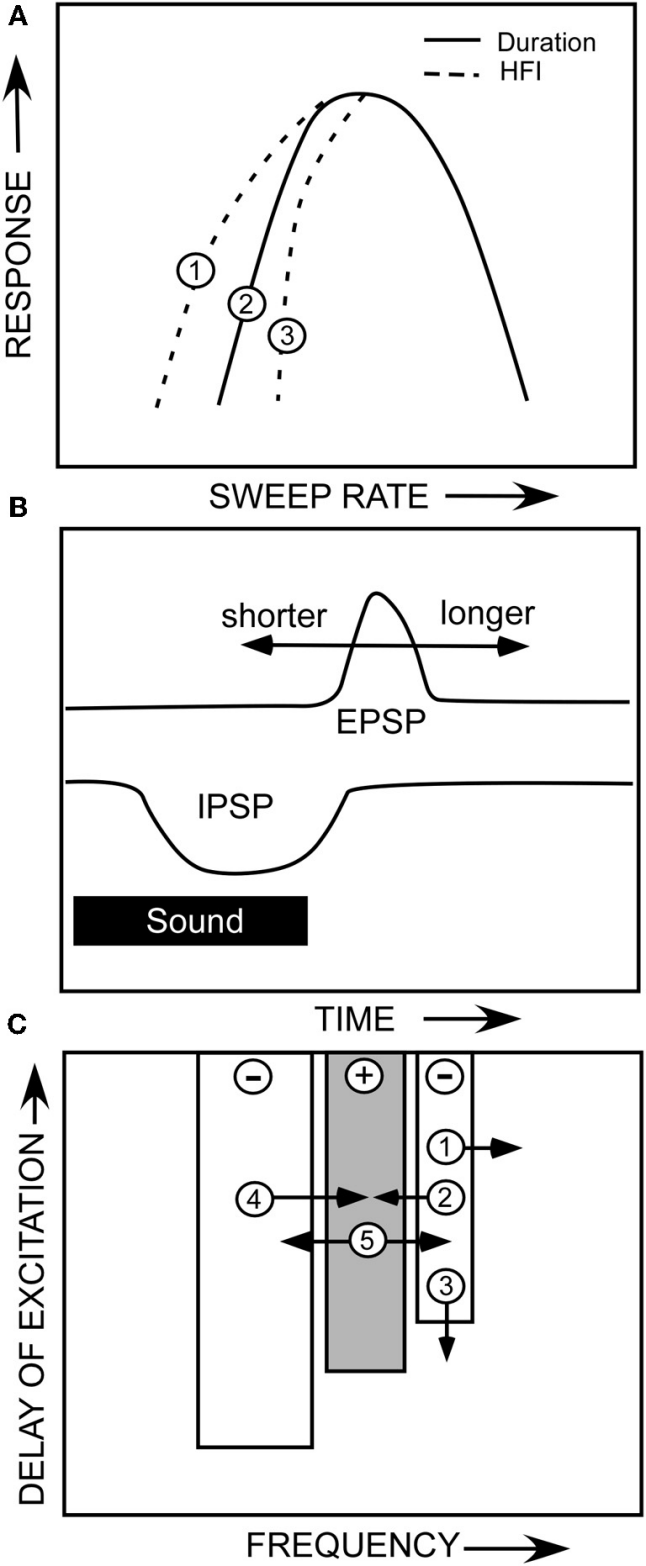
**(A)** A hypothetical neuron showing how duration tuning and HFI could potentially affect sweep rate selectivity. **(B)** The anti-coincidence mechanism thought to underlie bandpass duration tuning. **(C)** A diagram of the multiple ways that changes in the properties of a neuron's inhibitory and excitatory frequency domains could influence sweep rate selectivity. See text.

In 5 of 15 bandpass rate-selective neurons tested, blocking inhibition had little effect on duration tuning. The example in Figure 10 shows that blocking GABAergic inhibition elevated response magnitude but did not eliminate bandpass duration tuning (Figure 9A). Similarly, it had little effect on the arrival time of HFI (Figure 9B). Consequently, blocking inhibition did not eliminate bandpass rate selectivity in this neuron (Figure 9C).

Finally, we have emphasized the mechanisms that shape bandpass selectivity and the slowest sweep rates to which a neuron will respond. Regarding the fastest sweeps to which these neurons respond, it is important to note that blocking inhibition had little or no effect on this response property. All neurons stopped responding at increasingly short tone durations, or increasingly fast sweep rates, and this is likely due to the intrinsic properties of neurons in the circuits projecting to these IC neurons, rather than the inhibitory inputs they receive.

## Discussion

Present results demonstrate that similar expressions of bandpass FM sweep rate selectivity can be created by either bandpass duration tuning, or a delayed HFI that determines the slowest sweep rate to which a neuron will respond. The elimination of HFI by starting an FM sweep at a frequency lower than the HFI will eliminate bandpass rate selectivity. The elimination of duration tuning or HFI in these neurons through the blockade of inhibitory receptors will also eliminate rate selectivity. If a neuron possessed both duration tuning and HFI, both mechanisms had to be eliminated in order to eliminate a neuron's bandpass rate selectivity.

### Complementary or redundant?

That two mechanisms can create the same sweep rate selectivity begs the question of whether these mechanisms are redundant or complementary. Both are true in that both mechanisms can contribute to the rate selectivity of a given neuron, or either can act alone. As shown in the hypothetical neuron in Figure 10, duration tuning alone can create bandpass rate tuning (Figure 10A, solid line). This type of duration tuning has been modeled as a coincidence mechanism in which an excitatory rebound from the IPSP combined with the excitatory input to drive the neuron (Casseday et al., 1994; Covey et al., 1996; review, Sayegh et al., 2011). Alternatively, we have modeled the mechanism underlying duration tuning as an early inhibition that persists the duration of a tone, and a delayed excitatory input that has a fixed latency (Figure 10B). The neuron will respond to a tone of increasing duration until the inhibition overlaps with the delayed excitation, and suppresses the response, and has thus been termed an anti-coincidence model (Fuzessery and Hall, 1999). The best duration, and the range of excitatory durations, of a neuron will be determined by the arrival time of the excitatory input (Figure 10B). If the excitatory input is delayed further, the neuron will respond to longer tone durations. Conversely, if it arrives earlier, the neuron can respond only to shorter durations before it is inhibited. It is not clear what determines the arrival time of the excitatory input; we have previously reported (Fuzessery et al., 2002) that blocking inhibitory inputs to IC neurons in the pallid bat have little effect on their response latency.

If HFI is also present, it can directly or indirectly contribute to the sculpting of rate selectivity. The arrival time of HFI is influenced by two factors. The cutoff sweep rate can be predicted by the inhibitory bandwidth (kHz) divided by the arrival time (ms). The broader the bandwidth of the inhibitory sideband (Figure 10C,1), the sooner a downward sweep will encounter the sideband and trigger inhibitory input. Also, if HFI simply arrives sooner relative to excitation (Figure 10C,3), the cutoff rate will be faster. If the cutoff rate shaped by HFI is faster than that shaped by the duration function (Figure 10A,3), it will directly contribute to shaping the slow-rate flank of the bandpass rate function (e.g., Figure 5C). If it is not faster (Figure 10A,1), it will not contribute, and the rate function will be shaped entirely by the duration function.

Even if HFI does not directly shape rate selectivity, it can have an indirect influence. If a neuron's rate selectivity is shaped by duration tuning, its best rate can be predicted by the bandwidth of its excitatory tuning curve (kHz) divided by its best duration (ms). Inhibitory sidebands can shape this excitatory bandwidth (Yang et al., 1992; Fuzessery and Hall, 1996; LeBeau et al., 2001), as shown in Figures 8B,E, where disinhibition eliminates inhibitory sidebands and expands the excitatory tuning curve. If strong inhibitory flanks narrow the excitatory bandwidth (e.g., Figure 10C,2,4), the best rate will decrease, assuming that the best duration remains constant. Conversely, if the excitatory bandwidth increases (Figure 10C,5), the best rate will increase because an FM sweep now has to traverse the broader excitatory tuning curve more rapidly. For example, if a neuron's best duration is 1 ms, and the excitatory bandwidth was to increase from 3 to 6 kHz, the best rate would increase from 3 to 6 kHz. There are thus multiple ways in which duration tuning and HFI can interact directly and indirectly, in a redundant or complementary fashion, to shape selectivity for FM sweep rate.

Asymmetrical facilitation is a third mechanism that is known to shape sweep rate selectivity in the pallid bat IC (Williams and Fuzessery, 2010). It has been most thoroughly documented in the FM specialists because the weak response to single tones makes the two-tone facilitation readily apparent. FM specialists were not included in the present study because strong responses to tones were required to predict sweep rate functions. The extent to which asymmetrical facilitation contributed to rate selectivity in this study is not clear, but our ability to predict rate selectivity, and to also eliminate it by eliminating duration tuning or HFI, suggests that this third mechanism did not play a major role in shaping the selectivity of the neurons tested in the present study.

### Circuitry underlying the creation of sweep rate selectivity

Inhibitory circuits play a significant role in shaping duration tuning, HFI, and FM sweep selectivity in the pallid bat IC (Fuzessery and Hall, 1996, 1999), so the present finding (Table 2) that duration tuning persists in 8 of 15 neurons, and HFI persists in 12 of 15 neurons in which inhibitory inputs were blocked suggests that the pallid bat IC may be inheriting some of this response selectivity from lower levels of the system, rather than creating it within the IC. There was not a significant difference in the percentage neurons in which duration tuning was eliminated by GBZ or STRYCH, suggesting that both inhibitory circuits play a similar role in shaping this form of selectivity. HFI was eliminated in only 3 of 15 neurons, suggesting that this response property is largely inherited from lower levels. The issue of where this selectivity is created was more thoroughly addressed in a recent study of the pallid bat IC (Williams and Fuzessery, 2011) that focused on the creation of HFI and LFI, the latter shaping sweep direction selectivity. The application of GBZ or STRYCH eliminated LFI in the majority of neurons, suggesting that both inhibitory circuits can shape LFI. When both drugs were applied, LFI was eliminated in 86% of neurons, suggesting that much of the LFI, and therefore sweep direction selectivity, is created through intrinsic IC processing. In contrast, only STRYCH application eliminated HFI, and this occurred in only 33% of neurons. Even when both GBZ and STRYCH were applied, HFI was lost in only 38% of neurons. These results, along with those of the present study, are consistent in that they both suggest that the HFI inhibition shaping rate selectivity is largely inherited from lower levels of the system. These results also suggest that GABA- and glycinergic circuits may play differential roles in shaping various forms of response properties in the auditory system.

### Underestimation of sweep rate selectivity

When predicting a neuron's sweep rate function from its duration function, there was an overall underestimation of rate selectivity by a factor of 1.6. In other words, although the shape of the predicted rate function closely approximated the actual function, it was shifted toward lower sweep rates. This is likely due to an underestimation of the bandwidth of the excitatory frequency domains. Auditory neurons have been found to respond to frequencies outside of the excitatory tuning curve, which is typically described using tones, if those frequencies are presented as part of a spectrotemporally more complex sound. This has been referred to as their “extraclassical” tuning curve (Xie et al., 2007; Schneider and Woolley, 2011). The pallid bat IC contains neurons termed FM specialists that respond preferentially or exclusively to downward FM sweeps that mimic its biosonar signal (Fuzessery, 1994). A large percentage of these exhibit an asymmetrical facilitation when presented with two tones delayed in time, as they would occur within a downward (but not upward) FM sweep (Williams and Fuzessery, 2010). When the excitatory domains of these FM specialists were measured with a series of narrowband downward FM sweeps, the bandwidths of the excitatory domains were broader than when measured with tones. We therefore suggest that some predicted rate functions were underestimated because we used tones to measure the excitatory tuning curve, but obtained sweep rate functions using FM sweeps. The FM sweeps excited the broader extraclassical tuning curve, but the tones did not. Since the predicted rates equal the excitatory bandwidth divided by the best duration, this would result in a lower predicted sweep rate.

### Behavioral relevance of duration and rate tuning

Duration selectivity has been found in both echolocating mammals (Ehrlich et al., 1997; Galazyuk and Feng, 1997; Fuzessery and Hall, 1999; Casseday et al., 2000; Faure et al., 2003; Mora and Kossl, 2004; Fremouw et al., 2005; Jen and Wu, 2006; Luo et al., 2008; Macias et al., 2011) and non-echolocating mammals (Chen, 1998; Brand et al., 2000; Xia et al., 2000; He, 2002; Perez-Gonzalez et al., 2006; Wang et al., 2006), and in anurans (Potter, 1965; Narins and Capranica, 1980; Gooler and Feng, 1992; Leary et al., 2008; Xia et al., unpublished data). These neurons likely play important roles in the selective detection and analysis of communication signals and biosonar echoes (review, Sayegh et al., 2011). Several forms of duration tuning have been reported in the IC, including allpass, shortpass, longpass, bandpass, and even band-reject. In the present study, we reported only bandpass and longpass because we sampled responses over a broad range of durations, including the microsecond range. This eliminated the allpass and shortpass categories, since there is a minimum sound duration required by all neurons to elicit a response.

To our knowledge, it is only in the pallid bat where the idea has been suggested that bandpass duration tuning serves to create FM sweep selectivity, and not to detect signal duration *per se* (Fuzessery et al., 2006; review, Sayegh et al., 2011). Indeed, such neurons are sweep-rate selective, because even when an FM sweep's bandwidth or duration is changed, these neurons continue to maintain the same best sweep rate (Fuzessery et al., 2006). It is axiomatic that a neuron that has bandpass duration tuning will also have bandpass sweep-rate selectivity. In other words, what is a purely temporal filter when presented with a tone becomes a spectrotemporal filter when presented with a spectrotemporally dynamic sound.

Although we initially reported (Fuzessery and Hall, 1999) that the durations of echolocation pulses (1.5–6 ms) roughly matched the range of best durations (0.5–7 ms), the match between the best sweep rates and echolocation pulse sweep rates is closer. Pallid bat echolocation pulses have bandwidths of 20–30 kHz (Brown, 1976; Fuzessery et al., 1993), which over the range of pulse durations would yield sweep rates of 3–20 kHz/ms. The best sweep rates of IC neurons range from 1 to 10 kHz, with a mean of 4 kHz/ms (Fuzessery et al., 2006). Although the distributions do not overlap perfectly, most rate-tuned neurons will respond to the range of sweep rates. If durations are considered, the mean best duration of IC neurons is 1.6 ms, which means almost half of the population is tuned to durations shorter than the minimum 1.5 ms pulse duration. We therefore suggest that, during development, the system tunes itself to the sweep rate of the echolocation pulse (and not its duration), since this is the primary signal that it is responsible for processing. This may also apply to other bat species, but interestingly, most of the other bat species that have been studied have constant-frequency (CF) or quasi-CF biosonar pulses (Casseday et al., 1994; Luo et al., 2008; Macias et al., 2011). Since these signals are essentially tones or shallow FM sweeps, a neuronal tuning to duration rather than sweep rate has greater behavior relevance. Perhaps the common denominator among these bat species is the need for the selective detection of echoes. In the pallid bat, we have suggested (Fuzessery, 1994) that neuronal selectivity for sweep rate is one of several filters, along with sweep direction and spectrum selectivity, that serve the selective detection of biosonar echoes by eliminating responses to other sounds in the environment. This function may be particularly important in a gleaner like the pallid bat, which uses echolocation primarily for general orientation, while passively listening for prey-generated sounds. Strong spectrotemporal filters may facilitate the segregation of these two auditory streams while the bat is hunting (Fuzessery, 1994; Barber et al., 2003).

Similar bandpass selectivity for short signal durations is also present in the lateral IC of the pallid bat (Fuzessery, 1997; Fuzessery and Hall, 1999). This region is tuned to frequencies below the echolocation pulse, and its neurons respond preferentially to noise transients used in the passive sound localization of prey (Bell, 1982; Fuzessery et al., 1993). This raises the interesting possibility that, while the duration tuning in the two neuronal populations is similar, one population may be tuned to the sweep rate of biosonar echoes, while the other is selective for the duration of short noise bursts.

To conclude, the ecological niche ocuppied by the pallid bat appears to necessitate the participation of neurons that respond selectively to biologically relevant signals, one of them being biosonar echoes. Selective pressure has apparently acted to produce multiple mechanisms that act separately or in concert to create the required degree of selectivity. Within this context, it is of interest that the two mechanisms discussed here appear to be the result of different inhibitory circuits within the auditory brainstem. The on-best frequency inhibition shaping duration tuning is either GABA- or glycinergic, while HFI is primarily the result of only glycinergic input (Williams and Fuzessery, 2011), suggesting that selective pressure has acted upon the two inhibitory pathways in a differential manner.

### Conflict of interest statement

The authors declare that the research was conducted in the absence of any commercial or financial relationships that could be construed as a potential conflict of interest.
